# Clinical Biochemistry of Serum Troponin

**DOI:** 10.3390/diagnostics14040378

**Published:** 2024-02-09

**Authors:** Ilhan Gokhan, Weilai Dong, Daniel Grubman, Kenechukwu Mezue, David Yang, Yanting Wang, Parul U. Gandhi, Jennifer M. Kwan, Jiun-Ruey Hu

**Affiliations:** 1 Yale School of Medicine, Yale University, New Haven, CT 06510, USA; ilhan.gokhan@yale.edu (I.G.);; 2 Section of Cardiovascular Medicine, Yale School of Medicine, Yale University, New Haven, CT 06510, USAjennifer.kwan@yale.edu (J.M.K.); 3 Department of Emergency Medicine, Yale School of Medicine, Yale University, New Haven, CT 06510, USA; 4 Division of Cardiovascular Disease and Hypertension, Rutgers Robert Wood Johnson Medical School, New Brunswick, NJ 08901, USA

**Keywords:** troponin, angina, biochemistry, high-sensitivity assay, conditional probability

## Abstract

Accurate measurement and interpretation of serum levels of troponin (Tn) is a central part of the clinical workup of a patient presenting with chest pain suspicious for acute coronary syndrome (ACS). Knowledge of the molecular characteristics of the troponin complex and test characteristics of troponin measurement assays allows for a deeper understanding of causes of false positive and false negative test results in myocardial injury. In this review, we discuss the molecular structure and functions of the constituent proteins of the troponin complex (TnT, TnC, and TnI); review the different isoforms of Tn and where they are from; survey the evolution of clinical Tn assays, ranging from first-generation to high-sensitivity (hs); provide a primer on statistical interpretation of assay results based on different clinical settings; and discuss potential causes of false results. We also summarize the advances in technologies that may lead to the development of future Tn assays, including the development of point of care assays and wearable Tn sensors for real-time continuous measurement.

## 1. Introduction

Troponins (Tn) are a group of regulatory proteins localized to the myofibrillar apparatus, which regulate excitation-contraction coupling, and are released into the bloodstream during myocardial injury. Accurate measurement of serum Tn levels plays a central role in the evaluation of patients presenting with chest pain concerning for acute coronary syndrome (ACS). Since the etiology of this symptom includes both benign and life-threatening etiologies, expeditious workup is crucial and calls for the availability of reliable assays. Over the past several decades, methods for Tn measurement have become increasingly precise, sensitive, and rapid. In this review, we summarize the molecular structure of the Tn complex, describe its different isoforms, survey first- through fourth-generation and high-sensitivity Tn (hs-Tn) assays, and provide a biostatistical background to understand its interpretation. In the accompanying clinical companion to this review [1], we highlight important clinical considerations of Tn assays, including cardiovascular and non-cardiovascular etiologies of Tn elevation.

## 2. Molecular Structure of Troponins

**Figure 1:**
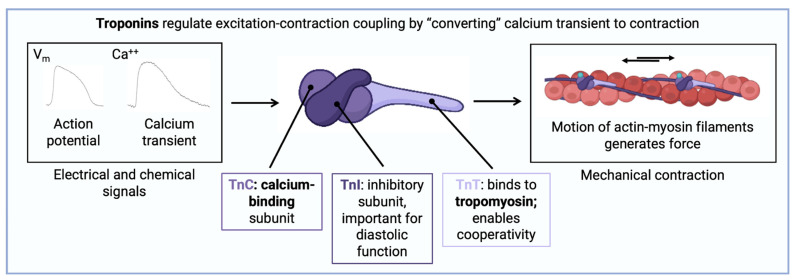
Structure and function of the troponin C (TnC), troponin I (TnI), and troponin T (TnT) components of the troponin complex and their roles in generating mechanical contraction with actin and myosin.

Troponins are a class of proteins that play a master regulatory role within the sarcomeres of striated muscle. Owing to their importance, they are evolutionarily conserved, and first appeared in organisms 250 million years ago, before mammalian and avian evolution diverged [2]. The Tn complex is a heterotrimer consisting of troponin C (TnC), troponin T (TnT), and troponin I (TnI) subunits, which associate closely with tropomyosin on the thin filament (actin) [3] (Figure 1). Based on the sliding filament theory, muscle contraction involves interaction between actin (the thin filament) and myosin (the thick filament); myosin exhibits ATPase activity that propels the motion of interdigitated thick and thin filaments past one another through a conformational change in protein structure known as the power stroke [4]. Interaction between actin and myosin is inhibited by the protein tropomyosin and promoted by surges of cytosolic calcium [5]. By virtue of their localization on the thin filament, and interactions with tropomyosin, Tn are well-poised to modulate muscle function. 

Fundamental discoveries about the Tn complex were made in the 1970s and 1980s using classical experimental techniques that isolated its constituents using protein purification [6]. Further work revealed that TnC is a calcium-binding protein that responds to increases in cytosolic levels [7]; that TnI is an inhibitory molecule that interferes with actomyosin binding [8]; and that TnT binds to tropomyosin and regulates its position relative to actin [9]. 

The first component of the Tn complex to be structurally characterized was TnC. Using X-ray crystallography, and later Nuclear Magnetic Resonance spectroscopy, it was determined that TnC has two domains connected by a flexible linker, each containing two calcium binding sites [10]. When the N-terminal regulatory domain (NTnC) binds calcium, a hydrophobic patch is revealed, enabling NTnC to bind to the switch region of TnI [11]. Consequently, TnI is relieved of its actin inhibition, so cross-bridge cycling can proceed and muscle contraction occurs. Structural work on TnC is notable because several small molecules, most notably levosimendan, have been shown to bind this protein to increase its sensitivity to calcium, and may be a possible therapeutic target in decompensated heart failure [11].

TnI, in contrast to the bi-domain structure of TnC, is a globular protein that binds to several components of the Tn complex: TnC, TnT, tropomyosin, and actin [12,13]. These numerous interactions, some of which are redundant, allow TnI to prevent actomyosin cross-bridge cycling under conditions of low calcium. A notable feature of TnI is the presence of multiple sites for post-translational modifications, which can tune its function based on physiological needs. Classic experiments in Langendorff-perfused rabbit hearts have demonstrated this. For example, inotropic stimulation of an ex vivo rabbit heart causes rapid phosphorylation of TnI at sites S23 and S24 by protein kinase A (PKA). This phosphorylation decreases the calcium sensitivity of TnI, promoting faster relaxation. Rapid relaxation during an adrenergic stimulus is advantageous as it allows the myocyte to prepare for the next contraction [14,15]. Meanwhile, phosphorylation of TnI at sites S43 and S45 by protein kinase C has the opposite effect, slowing the myosin ATPase rate and increasing the calcium sensitivity of TnI [16,17]. In contrast to TnC, which is an important regulator of contractility, TnI can be seen as a primary regulator of lusitropy. 

TnT is the largest and most complex protein of the Tn heterotrimer. It forms extensive interactions with TnI, TnC, tropomyosin, and actin [5]. TnT confers cooperativity to the interaction between the myosin ATPase and the actin thin filament [18]. It can also promote inhibition of contraction by strengthening the interaction between actin and tropomyosin. 

## 3. Identity and Source of Troponin Complexes

Within the cardiomyocyte, troponins are primarily localized to the sarcomere, which forms the myofibrillar apparatus, with only a small fraction in the cytosol, estimated at 3.5% and 7.0% of total TnI and TnT by mass, respectively [19]. During a myocardial infarction, TnI is released as a single peak, while TnT is usually released in a biphasic manner, with the second peak occurring approximately 80 h after the onset of chest pain, regardless of reperfusion [20]. One hypothesis for this biphasic behavior is that cytosolic TnT is released immediately into the circulation following damage to myocytes, whereas myofibrillar TnT takes longer to be released [20]. However, alternative explanations such as differences in the half-life of immune-reactive TnT fragments cannot be ruled out [20]. A recent study which used advanced biochemical techniques including gel filtration revealed that following myocardial infarction (MI), much of detectable TnT is part of a ternary complex of TnI, TnT, and TnC, which progressively gets degraded to lower molecular weight species, and that a complex of TnI and TnC also exists alongside free TnT fragments [21]. However, other studies have found contrasting results with no clear consensus [22,23,24]. Further complicating the matter is that troponins are subject to proteolytic degradation after release, with matrix metalloproteinase-2 (MMP-2), calpain-1 and calpain-2 cleaving TnI, and MMP-2 and calpain-1 cleaving TnT [24,25]. Notably, knowledge of which fragments are found in the serum is useful for rationally designing antibodies against the most stable Tn epitopes, but it is possible that different Tn complexes are found in different disease states. A fascinating prospect suggested by preliminary data, which has yet to be realized, is the discovery of disease-specific Tn complex isoforms, which could enable the differentiation of the etiology of Tn elevation and lead to more specific diagnoses [26,27,28].

There are many mechanisms for Tn release into the serum after cardiomyocyte injury. The most common mechanism is myocyte necrosis, when myocytes undergo necrosis due to ischemic injury—for example, Ca^2+^ leaks into the cytoplasm, which causes massive contraction of all sarcomeres and subsequent consumption of all ATP, resulting in the total destruction of cell contents [29]. However, apoptosis can also be responsible for Tn release: in a porcine model of brief (10 min) coronary occlusion, which is not long enough to cause necrosis or infarction, significant amounts of TnI were released into the serum from apoptotic cells [30]. Even transient increases in left ventricular end-diastolic pressure using a model of reversible left ventricular dysfunction can cause stretch-induced myocardial stunning, apoptosis, and Tn release [31]. Finally, it is even possible to have Tn release from living cells by mechanisms including ischemia-induced membrane blebbing, a stretch-induced integrin response, cell turnover, and inflammation [32,33].

## 4. Isoforms of Troponin

**Figure 2:**
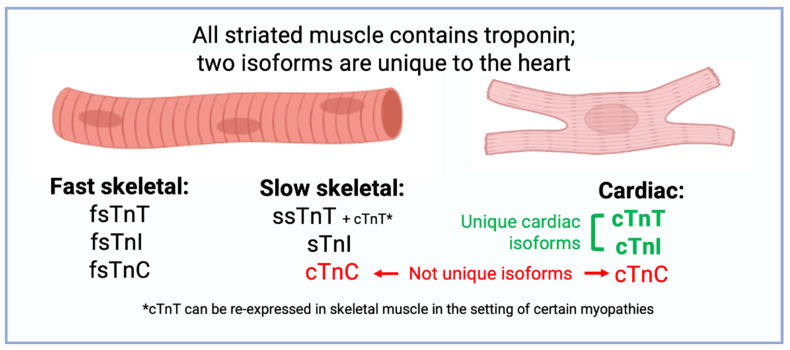
Isoforms of troponin and their location in the human body.

TnC has only two isoforms in the human genome: cardiac troponin C (TnC, gene name *TNNC1*) and fast-twitch skeletal TnC (fsTnC, gene name *TNNC2*) (Figure 2). TnT has a molecular weight of 18 kDa and is composed of 210 amino acids that are not alternatively spliced [34]. The isoforms of TnC expressed in cardiac and slow-twitch skeletal muscle are identical, while the isoform of TnC expressed in fast-twitch skeletal muscle is unique [35,36]. TnC and fsTnC have similar C-terminal domains which each bind two calcium ions, while TnC has only one calcium-binding site in its N-terminal domain which is necessary for calcium-induced cardiac muscle contraction [37]. Because the isoform of TnC expressed in cardiac muscle is identical to TnC expressed in slow-twitch skeletal muscle, measurement of TnC in a clinical setting would not be able to differentiate the etiology of muscle disease. Hence, commercial assays for clinical use focus on either TnI or TnT. 

TnI has three separate protein-coding genes: *TNNI1*, which encodes slow-twitch skeletal TnI (ssTnI); *TNNI2*, which encodes fast-twitch skeletal TnI (fsTnI); and *TNNI3*, which encodes cardiac TnI (Tn). TnI is the largest isoform, has a unique N-terminal sequence with PKA-regulated phosphorylation sites (S23 and S24) not seen in other TnI isoforms, and is not alternatively spliced [34]. The fetal heart expresses ssTnI, but by nine months of life only TnI is detectable at the transcript and protein level [38]. Though it was previously hypothesized that heart failure would result in re-expression of fetal ssTnI, immunoblotting and northern blotting refuted this hypothesis [38]. However, phosphorylation studies using antibodies against phospho-TnI reveal that in heart failure, the relative fraction of phosphorylated TnI decreases, suggesting a potential adaptive or maladaptive response to smaller calcium transients seen in the failing heart [39].

TnT has the most complex variety of isoforms relative to the other troponins. TnT has three separate protein-coding genes: *TNNT1*, which encodes slow-twitch skeletal TnT (ssTnT); *TNNT2*, which encodes cardiac TnT (TnT); and *TNNT3*, which encodes fast-twitch skeletal TnT (fsTnT) [34]. TnT encodes 17 exons, of which exons 4, 5, and 13 can undergo alternative splicing [40]. Gene products resulting from alternative splicing of exons 4 and 5 yield four cardiac-specific TnT proteins, with each sub-isoform having a unique calcium sensitivity and ability to inhibit myosin ATPase [41]. Though the biophysical basis of each isoform is beyond the scope of this review, several observations have particular clinical relevance: First, the N-terminal tail of TnT can be cleaved by μ-calpain in the setting of ischemia-reperfusion, which preserves stroke volume through an interaction with tropomyosin [42]. Second, an exon-4-skipped TnT isoform is overexpressed in the failing human heart, in familial hypertrophic cardiomyopathy, and in a diabetic rat heart [43]. Third, in the setting of skeletal muscle disorders, cardiac-specific TnT is often re-expressed in diseased skeletal muscle and detectable at the proteomic and transcriptomic levels [44,45]. This may confound the clinical interpretation of positive TnT tests among this population, which is discussed in detail in the clinical companion to this review [1]. 

## 5. Reference Limit Definitions

The limit of blank (LoB) represents the noise inherent to the measurement system, and is defined as the 95th percentile of the analytic signal when no Tn (0 ng/L), only plasma or serum, is present in samples tested [46]. The limit of detection (LoD) is an analytical parameter used to detect the minimal concentration of Tn obtained in the biologic fluid and is defined as the mean signal at which 95% of the frequency distribution is above the LoB. The LoD is always higher than the LoB, by definition. 

The limit of quantification (LoQ) shows the functional sensitivity of the Tn assay and is defined as the minimum concentration at which the coefficient of variation is ≤20% [47]. The LoQ is thus always higher than the LoD, by definition. Imprecision at the LoD is high, whereas measurements at the LoQ can be reported with a degree of certainty (CoV ≤ 20%). The LoQ is FDA-reportable, but not the LoD. It has been previously demonstrated that LoQ < 6 ng/L is a safe cutoff to rapidly rule out acute MI through a single hs-TnT test in the ED [48]. According to the 2021 AHA/ACC guidelines [47,49], for patients with acute chest pain, a normal ECG, and ACS symptoms that started at least 3 h prior to ED arrival, a single hs-Tn concentration that is below the LoD on the initial measurement (i.e., “undetectable”) can reasonably exclude myocardial injury, whereas patients with detectable concentrations should proceed with serial measurement of hs-Tn (e.g., at 1 and 3 h). The AHA/ACC guidelines do not endorse a single particular protocol for 0/1/3 h, 0/1 h, or 0/2 h rule-out, unlike the ESC guidelines [50].

The 99th percentile upper reference limit (URL) of Tn is used to support the diagnosis of acute myocardial injury or MI as recommended by multiple international committees, including the European Society of Cardiology, ACC, AHA, National Academy of Clinical Biochemistry, the International Federation of Clinical Chemistry Task Force and Committee of Clinical Applications of Cardiac Biomarkers [51]. It is defined as the concentration detected exceeding the 99th percentile of the values of a healthy reference population. It is approximately three standard deviations above the mean of the normally distributed population. It was originally adopted to match the cutoff for CK-MB and the 99th percentile cutoff was chosen to reduce the erroneous interpretation of patients with MI [52]. Furthermore, the imprecision (coefficient of variation) was suggested to be less than 10% at the 99th percentile, as better precision allows for more sensitive assay [53]. It should be noted that the 99th percentile URL is assay-specific and can vary by sex.

## 6. Generations of Troponin Assays

### 6.1. First Generation

**Figure 3:**
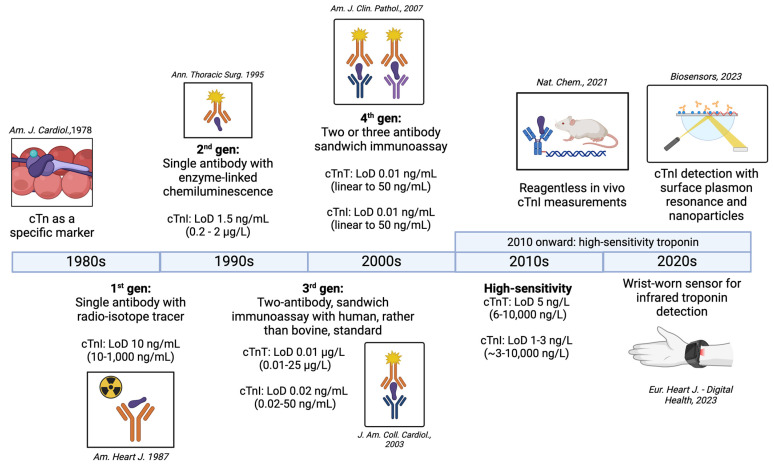
Evolution of troponin measurement assays from the 1980s to the 2020s, and the limit of detection (LoD) associated with each generation of assay. Major papers corresponding to each generation of troponin are referenced as [54,55,56,57,58,59,60,61], based on information from [62].

The first diagnostic assays for detecting Tn in serum were developed in the 1980s using radioimmunoassay technology (Figure 3). Prior to this, diagnosis of myocardial injury relied on markers such as creatine kinase-MB (CK-MB) and lactate dehydrogenase-1 and -2 (LDH-1/2). However, the expression of CK-MB and LDH is not unique to cardiac muscle, leading to diagnostic uncertainty. TnI was chosen for the first generation of Tn assays because its expression is limited to the myocardium. For this assay, antisera to cardiac TnI were raised in rabbits and sheep, serum samples were iodinated with [^125^I], and after precipitation of the antibody-TnI complex with an anti-rabbit or anti-sheep antibody, radioactivity was measured on a gamma counter. The entire procedure took between two and four days. The lower limit of detection was approximately 10 ng/mL, and the operating range was 10–1000 ng/mL. Cross-reactivity between cardiac and skeletal muscle proteins was estimated at <2% [54]. In an early study of 32 patients with acute MI, a 10–12-fold increase above normal TnI levels was detected, with a mean of 112 ng/mL (range 20–550 ng/mL), peaking between 15 and 24 h after MI [54].

### 6.2. Second Generation

The second generation of Tn assays had a level of detection that was an order of magnitude better than first-generation assays (~1.5 ng/mL) [55]. An important change was the introduction of direct enzyme-linked immunoassay (ELISA) technology in place of a radio-isotopic readout (Status E100 spectrophotometer, Kallestad Laboratories, Inc., Cheska, MN, USA). Measurements of TnI levels in sera were made using an optical readout of visible-light absorbance, which not only avoided exposure from the radioactive [^125^I]-labeling, but also afforded higher sensitivity, as the enzyme was able to amplify the signal from a minimal amount of Tn antigen [62]. Moreover, some second-generation assays began to use two Tn-specific antibodies to improve specificity and sensitivity. A typical second-generation assay, using ELISA technology, had a range from 0.2–20 μg/L and within-run coefficient of variation of 4.7%. In a notable improvement from first-generation assays, second-generation assays could be performed within 30 min and displayed no cross-reactivity with skeletal muscle [63]. A limitation of most second-generation TnT assays was that the calibration was performed with bovine TnT standards, which resulted in curves that were not always linear [64]. However, this limitation was addressed in third-generation Tn assays, described below.

### 6.3. Third Generation

Continued improvement in detection technology, including the Roche Elecsys 2010 immunology analyzer (Roche Diagnostics Corporation, Indianapolis, IN, USA), brought further improvements in sensitivity to third-generation assays. Crucially, this generation also adopted human Tn standards for calibration, eliminating previous issues with non-linearity encountered with bovine Tn standard use. For a typical third-generation TnT assay, the analytical range was approximately 0.01–25 μg/L, with 10% CoV at 0.03 μg/L. The URI, which represents the 99th percentile of a population of healthy controls, was below the lower LoD. In a typical laboratory setting, intra-assay and inter-assay CoVs were 7.9% and 11.2%, respectively [64]. As a result of the improved sensitivity, in a study of 750 patients admitted to a coronary care unit, 35% of additional patients were diagnosed with an acute MI [64]. Furthermore, being more sensitive, the third-generation assay was able to identify a subgroup of patients with minor myocardial injury, who nevertheless were found to have an increased risk of future cardiac events. The ability for mild Tn elevations to carry prognostic significance in subclinical disease would be a feature common to subsequent generations of Tn assays.

### 6.4. Fourth Generation

Fourth-generation assays continued to improve the sensitivity of Tn measurements compared to their third-generation counterparts. Most continue to use direct chemiluminescent technology which was introduced in the second-generation assays as described above. Notably, fourth-generation assays use at least two unique antibodies, with some using three antibodies. For example, one fourth-generation assay, the ADVIA Centaur TnI-Ultra, uses one monoclonal and two polyclonal antibodies raised against the central region of TnI, which allows for enhanced binding. When combined with a proprietary blocking reagent that removes nonspecific binding, this technique was able to achieve a [56] manufacturer-stated assay range of 0.006–50 ng/mL; the 99th-percentile value was 0.04 ng/mL and a 10% CoV was obtained at 0.02 ng/mL [56]. Another commonly used fourth-generation assay, the Roche Elecsys TnT Stat (Roche Diagnostics Corporation, Indianapolis, IN, USA), also uses direct sandwich immunoassay technology, but with streptavidin-coated microparticles to enhance the signal. The analytical sensitivity of this fourth-generation test is 0.01 ng/mL, with a concentration of 0.03 ng/mL providing a 10% CoV [65].

## 7. High-Sensitivity Troponin

### 7.1. Definition of High Sensitivity

The hs-Tn assays measure Tn levels using similar immunological methods to fourth-generation assays, but with the capability of detecting much smaller concentrations. The term “high sensitivity” does not refer to one specific technology for measurement or one specific form of Tn being measured but refers to any assay that meets two criteria. First, its total imprecision, as defined by the CoV, at the 99th percentile value, is ≤10%. Second, measurable concentrations below the 99th percentile should be attainable at a concentration value above the assay’s limit of detection for at least 50% of healthy individuals [66]. Concentrations of hs-Tn are expressed in nanograms per liter (ng/L) or picograms per milliliter (pg/mL). The high performance of hs-TnT assays has yielded a sensitivity >94% and a negative predictive value ≥99% for possible MI among patients in the emergency department (ED) setting [67]. However, such high sensitivity has implications for clinical practice and has also yielded new insights into the basic biology of cardiovascular disease.

### 7.2. Conceptualizing Equivalence of Fourth-Generation and High-Sensitivity Values

**Figure 4:**
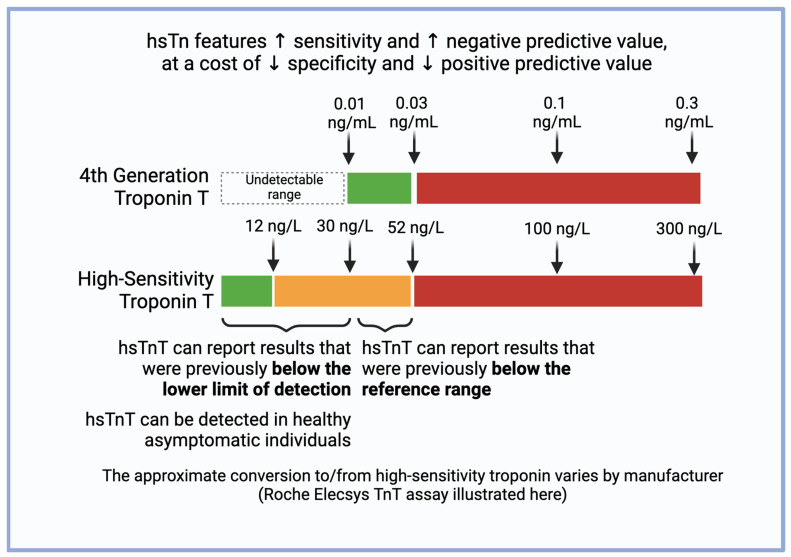
Approximate equivalence of fourth-generation (**top**) and high-sensitivity (**bottom**) troponin assays from one manufacturer (Roche). Hs-TnT assays can report results that were previously below the lower limit of detection on fourth-generation assays.

As of 2024, there are eight hs-Tn assays approved by the FDA, seven of which detect TnI and one of which detects TnT [47]. The LoD of these assays ranges between 1–3 ng/L; the LoQ, between 0.9–6 ng/L; and the overall 99th percentile, between 17.5 (Beckman Coulter Access two hs-TnI) and 60.4 (Siemens Dimension ExL TnI) [47]. The conversion of values from conventional to high-sensitivity assay varies by manufacturer. With the Roche Elecsys hs-TnT (Roche Diagnostics GmbH, Mannheim, Germany), for example, values above 100 ng/L on the hs-TnT assay correspond linearly and 1:1 to values above 0.1 ng/mL on the conventional assay, so a value of 300 ng/L on the hs-TnT assay corresponds to 0.3 ng/mL on the conventional assay [68]. Values below 100 ng/L on the hs-TnT assay have a non-linear relationship: 52 ng/L hs-TnT corresponds to 0.03 ng/mL, and 30 ng/L corresponds to 0.01 ng/mL (Figure 4) [69].

### 7.3. Clinical Implications of High-Sensitivity Troponin

Compared with conventional Tn assays, hs-Tn assays have higher negative predictive values for acute MI, meaning that a negative test result has a much higher certainty of truly representing the absence of acute MI. This reduces the “troponin-blind” interval, meaning that the time course for serial Tn checks can be shortened, resulting in a 4% absolute increase and 20% relative increase in the detection of type 1 MI, which consequently decreases the diagnosis of unstable angina due to the detection of abnormal Tn levels [50].

Because high-sensitivity assays can now detect serum Tn in an enlarged subset of patients without myocardial ischemia, clinicians must be more discerning when interpreting results. Compared with conventional Tn assays, hs-Tn assays carry a two-fold increase in the detection of type 2 MI [50]. Additionally, as highlighted in the accompanying companion review [1], many conditions other than ACS can present with elevations in serum Tn, including cardiac conditions such as tachyarrhythmia and noncardiac conditions such as pulmonary (e.g., pulmonary embolism), renal (end-stage renal disease), neurologic (e.g., stroke), musculoskeletal (e.g., rhabdomyolysis), oncologic (e.g., when undergoing certain chemotherapies), and GI causes, as well as trauma (e.g., blunt chest trauma) and acute illness (e.g., sepsis) [70,71,72,73]. The etiology of the elevation can affect treatment. For example, patients with Tn elevation due to sepsis would not benefit from antithrombotic medications and PCI, while those with ACS would [74]. The use of more than one (e.g., two) cutoff points may facilitate clinical reasoning, with a more sensitive cutoff being good for ruling out ACS in the emergency setting, and a more specific cutoff being good for ruling in ACS and guiding treatment. 

Furthermore, Tn levels between the LoQ and the 99th URL has been used in studies to identify patients at risk of coronary artery disease (CAD). One study showed that patients with intermediate Tn concentration (5 ng/L to the 99th URL) had a higher risk of MI and cardiac death in one year compared to patients who had a Tn concentration <5 ng/L (3.3% vs. 0.6%, *p* < 0.0001) [75]. Another study also suggested that patients with Tn of 5 ng/L to the 99th URL were three times more likely to have CAD and had a greater atherosclerotic burden as detected by coronary CT angiography compared to patients with Tn < 5 ng/L [76]. Thus, the increased sensitivity of Tn may allow it to play further roles in risk stratification, including with imaging.

### 7.4. Sex-Specific Considerations of High-Sensitivity Troponin

Notably, the distribution of hs-Tn values is not uniform across sexes. In both hs-TnT and hs-TnI assays, the 99th percentile URL in men is higher than the 99th percentile URL in women [77]. The same hs-TnT concentration may carry different prognostic implications in men and women. For example, in one study of 19,501 individuals, at 10 ng/L, the hazard ratio for the composite outcome of cardiovascular death, myocardial infarction, or stroke was 9.7 (95% CI 7.6–12.4) for women and 5.6 (95% CI 4.7–6.6) in men, relative to the limit of blank [78]. Use of sex-specific thresholds may increase the diagnosis of MI in women. Similarly, in a prospective cohort study of 1126 patients with suspected ACS, the use of sex-specific thresholds for hs-TnI substantially increased the diagnosis of MI in women (11% to 22, *p <* 0.001) and had a minimal effect on the diagnosis of MI in men (19% to 21%, *p* = 0.002) [79]. However, the increased diagnosis rate may not necessarily have an effect on prognosis. In SWEDEHEART, the use of sex-specific hs-TnI cutoffs in 12,489 patients increased incidence of acute MI by 11.5% (female) and 9.8% (male), but had no major effect on cardiovascular outcomes after multivariable adjustment (HR 0.91 [95% CI 0.80–1.03], *p* = 0.126) [80]. Further research is needed on the role of sex-specific strategies for hs-Tn-based diagnosis and management.

## 8. Future of Troponin Assays

With recent advances in computation, there are several ways that currently used troponin tests can be incorporated within novel algorithms to yield better clinical performance. For example, the MI3 (Myocardial Ischemic-Injury Index) algorithm, which uses gradient-boosting machine learning, was shown to perform better than the ESC 0/3-hr pathway when tested on a cohort of 404 patients with symptoms concerning for ACS who had sequential hs-TnI measurements [81]. This was replicated in a larger cohort of more than 20,000 patients with symptoms concerning for ACS, in which the stratification of patients into low- and high-probability cohorts yielded a negative predictive value of 99.8% and a positive predictive value of 70.4%, respectively [82]. However, other algorithms seek to complement hs-TnI measurements with other biomarkers to differentiate between Type 1 and Type 2 MI. In this prospective trial of 748 patients presenting with suspected MI, the measurement of three additional biomarkers (BNP, copeptin, and apo A-II), yielded an area under the ROC of 0.82 for the differentiation of Type 1 and Type 2 MI [83]. Therefore, the potential of novel algorithms to enhance interpretation of currently used troponin assays has yet to be fully tapped.

Besides the aforementioned advances in computation that have improved interpretation of current troponin tests, several advances in basic science and engineering have made possible an emerging generation of Tn assays that may have the potential to perform better than current high-sensitivity assays. One such technology, called surface plasmon resonance (SPR), has been used extensively to characterize interactions between biomolecules by measuring the refractive index of polarized light impinging on a metal film [84]. SPR affords high sensitivity, real-time detection, and label-free quantification of biomolecules, making it an attractive platform for measuring Tn. One such platform, using a nano-imprinted molecular polymer chip, affords a lower limit of detection of 0.53 ng/mL, with a linear detection range of 0.78–50 ng/mL [57]. Although the sensitivity of this assay is lower than that of hs-TnI assays in clinical use, it is a promising demonstration of SPR technology. A similar platform using gold nanoparticles affords a sensitivity of 0.015 ng/mL, with results reportable in less than 15 min—all requiring a sample volume of only 2 μL [85]. Finally, an assay that uses gold nanoparticles (pGold) in conjunction with a near-infrared light source was able to achieve an LoD comparable to hs-Tn assays in clinical use (3 ng/L). Notably, when this assay was used on a cohort of 112 patients with MI, it yielded an area under the curve (AUC) of 0.976—comparable to that of an hs-TnI assay in clinical use (0.994)—while using twenty times less sample (200 μL vs. 10 μL) [86]. Therefore, next-generation detection technologies, such as surface plasmon resonance, have already reached the benchmark set by traditional (chemiluminescent) detection. 

The Tn assays mentioned thus far all require a serum sample in which to measure the concentration of the analyte. However, a new frontier in biomarker measurement involves in vivo measurements, which do not require a blood sample. By taking advantage of a molecular technology known as a “molecular pendulum”, which uses an antibody coupled to a DNA linker, whose activity is measured by a redox reaction, analyte levels could be measured in mouse blood, sweat, tears, saliva, and urine [58]. Though the application of this technique is currently limited to an in vivo model of doxorubicin-induced cardiotoxicity, the linear range of detection is as low as 100 pg/mL (100 ng/mL), and concentrations as low as 1 pg/mL can be detected. This is comparable to hs-Tn assays in clinical use and does not require a blood draw. 

In addition to the technology described above, which is currently limited to the research lab, a new generation of wearable Tn sensors has also been developed. These technologies seek to improve the sensitivity of point-of-care Tn assays, which often have insufficient sensitivity to rule out ACS. Recently, a technique known as mid-infrared attenuated total reflection spectroscopy has enabled noninvasive measurements of TnI through the skin, achieving a sensitivity of 96.3% and a specificity of 60% for predicting elevated vs. non-elevated Tn, with the gold standard being an hs-TnI assay (Advia Centaur, Siemens Healthineers, Milan, Italy) [59]. Moreover, in a prospective trial of 238 patients diagnosed with MI by conventional means, the transdermal, noninvasive technology yielded an AUC of 0.90–0.92 and also predicted the presence of significant coronary stenosis (OR 4.69, 95% CI 1.27–17.26, *p* = 0.019) [87]. Whether these emerging technologies reach the market in the coming years will depend on further validation studies in diverse populations and regulatory clearance.

## 9. Conditional Probability for the Interpretation of Troponin Testing

It is crucial to avoid treating elevations in serum Tn as a binary classification of “positive” or “negative” based on a single cutoff. Appropriate interpretation of elevations in serum Tn requires an understanding of conditional probabilities, which are in turn based on the clinical context and the characteristics of the assays. Conditional probability describes the way the probability of an event (presence of acute MI) is changed by the knowledge of previous events (Tn elevated). The fundamentals of conditional probability and how we notate it can be found below.

P(X) indicates the probability of event X. If A and B are independent events, the probability of A and B both happening, notated P(A^B), is P(A) × P(B). P(A|B) indicates the probability of A given B, which can be calculated as P(A^B)/P(B). When A and B are independent,

P(A|B) = P(A) × P(B)/P(B) = P(A).(1)

Meanwhile, positive predictive value (PPV) and negative predictive value (NPV) are used to express the clinical relevance of a test. PPV is the probability of truly having the disease given a positive test and is the fraction of true positives (TP) over the sum of the true positives and false positives (FP). NPV is the probability of truly having no disease given a negative test and is the fraction of true negatives (TN) over the sum of the true negatives and false negatives (FN).

PPV = P(disease|positive test)(2)

PPV = TP/(TP + FP)(3)

NPV = P(no disease|negative test)(4)

NPV = TN/(TN + FN)(5)

Sensitivity is the probability a patient with a disease will test positive and is a fraction of the true positives over the sum of the true positives and false negatives. Specificity is the probability a patient with a disease will test negative and is a fraction of the true negatives over the sum of the true negatives and false positives.

Sensitivity = TP/(TP + FN)(6)

Specificity = TN/(TN + FP)(7)

**Figure 5:**
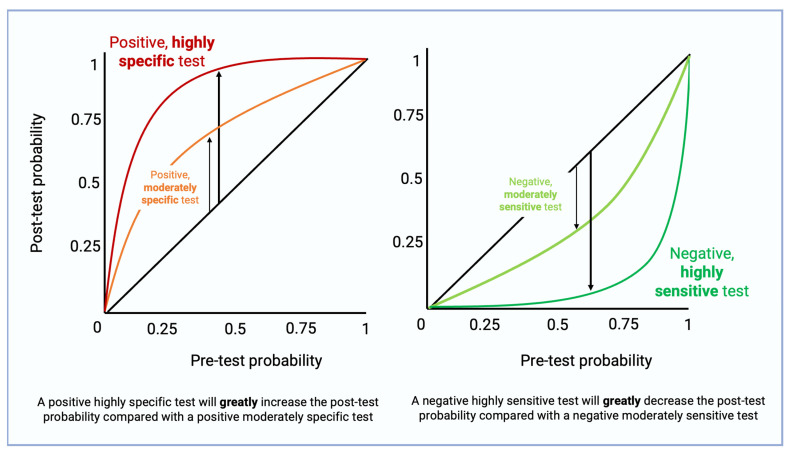
For a given pre-test probability, use of a highly specific test will greatly increase the post-test probability of disease compared to a moderately specific test, if the test result is positive. For a given pre-test probability, use of a highly sensitive test will greatly decrease the post-test probability of disease compared to a moderately sensitive test, if the result is negative.

Raising the cutoff value will increase specificity in exchange for poorer sensitivity. Conversely, lowering the cutoff value (or coming up with a new hs-Tn assay, for example) will increase sensitivity in exchange for poorer specificity. A highly sensitive test that is positive will greatly decrease the post-test probability compared with a moderately sensitive test that is positive (Figure 5). Given the increasing clinical use of hs-Tn, it is increasingly important to consider the complete clinical context, making use of electrocardiographic, echocardiographic, interview, and physical exam data to understand the pre-test and post-test probability of MI. Clinical sensitivity, which refers to the ability of an assay to correctly identify patients with the disease of interest, should be differentiated from analytical sensitivity. It is usually shown as LoD or LoQ, which shows the lowest concentration an assay can detect.

Likelihood ratios (LR) are used to express the capacity of a test. They are defined as the percentage of diseased patients with a given test result divided by the percentage of well people with the same test results [74]. Specifically, LR+ is the odds that a positive test means the patient truly has the disease, while LR− is the odds that a negative test means the patient truly does not have the disease. A high LR+ suggests the test is highly specific (odds are our positive test is a TP), and a low LR− suggests it is highly sensitive (negative values are rarely FN). Physical exam findings and elements of history can also carry LRs.

LR+ = sensitivity/(1 − specificity)(8)

LR− = (1 − sensitivity)/specificity(9)

LR can be used to directly calculate PPV and NPV based on pretest probability.

PPV = Pretest odds × LR (+)(10)

NPV = Pretest odds × LR (−)(11)

LR involves odds, not probabilities, but it can be used to calculate probabilities as follows:

Odds = (probability)/(1 − probability)(12)

Pretest odds × LR = post-test odds(13)

Probability = (odds)/(1 + odds)(14)

**Figure 6:**
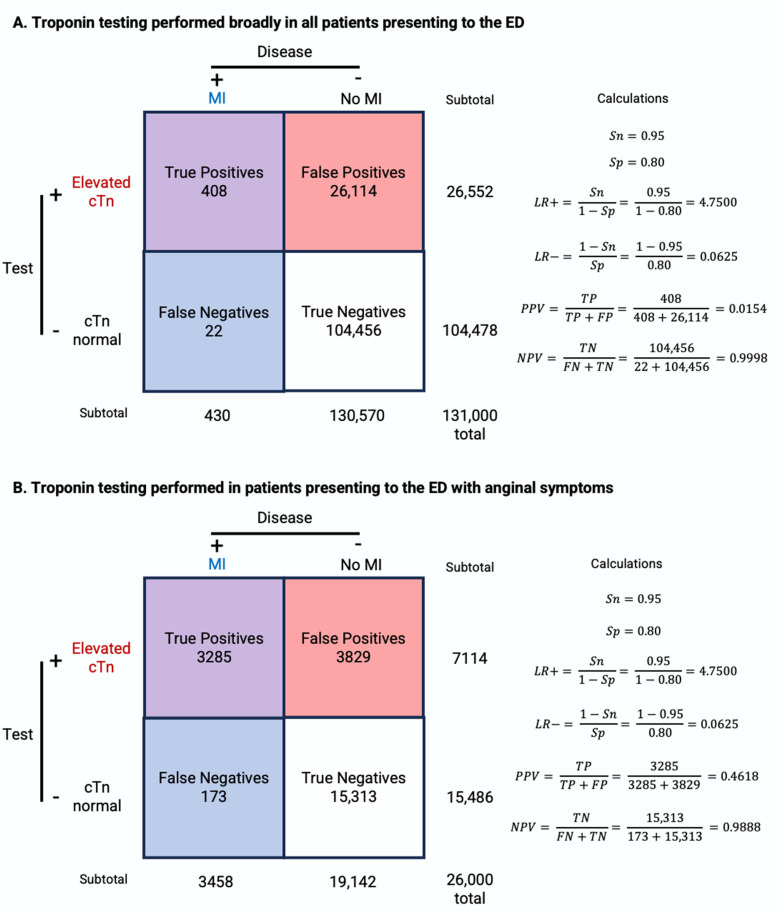
Two 2 × 2 tables and calculations of the positive and negative likelihood ratio (LR+, LR−) and positive and negative predictive value (PPV, NPV). In the top example, (**A**) troponin testing is performed broadly in all patients presenting to the ED, while in the bottom example, (**B**) troponin testing is performed selectively in patients who present to the ED with chest pain concerning for ACS. The PPV and post-test probability change dramatically (1.54% -> 46%) based on the pretest probability in the populations we are testing.

By applying the above statistical principles, we can interpret the Tn testing results under different clinical settings. In the following pair of numeric examples, we will examine the case of a patient who presents to the ED without chest pain and a patient who presents to the ED with chest pain, using real-world prevalence. In the first scenario, a patient presents to the ED with a complaint unrelated to chest pain, such as a headache or skin rash. If Tn testing is carried out on this individual, and it is found to be abnormal, what are the chances he has an acute MI given no other information? Assuming the prevalence of MI among the 131 million patients presenting to the ED annually is 0.328% (430,000) [88] and the Tn assay has a sensitivity of 95% and a specificity of 80% [89], PPV can be calculated as TP/(TP + FP) = 408/26,522 = 0.0154 = 1.54% (Figure 6A). Similarly, the post-test odds = pretest odds × LR = 0.00328/(1 − 0.00328) × 0.95/(1 − 0.8) = 0.0156 and post-test probability = post-test odds/(1 + post-test odds) = 0.0156/(1 + 0.0156) = 0.0154 = 1.54%. This means that even if the Tn test returns abnormal in this patient without chest pain, the likelihood that the abnormal test truly represents abnormal disease is low, at 1.54%. This illustrates that a strategy of broadly testing all patients who walk into the ED would provide us with little useful information and may lead to unnecessary downstream costs and harm to the patient.

On the other hand, if a patient presents to the ED describing chest pain concerning for ACS, and Tn testing is sent on this individual, what is PPV or post-test probability using the same assay? The prevalence of MI in a population of 22,600 patients with symptoms suggestive of MI is 15.3% [90], significantly higher than the 0.328% in the prior example. Thus, PPV = 3285/7114 = 0.462 = 46.2% (Figure 6B). Similarly, the post-test odds = 0.153/(1 − 0.153) × 0.95/(1 − 0.8) = 0.858 and post-test probability = 0.858/(1 + 0.858) = 46.2%. Now that we are specifically performing Tn testing on patients with suspected MI, a positive result is more meaningful. In this second scenario, the likelihood that the abnormal test truly represents abnormal disease (i.e., an MI) is 46%, significantly higher than the 1.54% in the prior example.

The above examples demonstrate the phenomenon of spectrum bias [91], which describes how the efficacy of the assay may vary when applied in different clinical settings and patient groups. In the above examples, the PPV and post-test probability changed dramatically (1.54% -> 46%) based on the pretest probability in the populations being tested. Studies designed to identify cutoffs for various diagnostic tests can run into issues where the selection of patients skews what is considered “normal” based on the number of diseased patients included and whether a case-control design was utilized (especially for rare conditions). Table 1 illustrates how the diagnostic characteristics of a test vary with respect to the prevalence of the disease in the population being tested. Advancing generations of Tn assays are better able to identify low-risk patients with minor myocardial necrosis [64]. Moreover, hs-Tn assays demonstrate higher sensitivity compared to conventional assays at the cost of specificity and precision. A meta-analysis including 17 studies showed that hc-Tn has a significantly higher sensitivity of 88.4% compared to 74.9% of conventional Tn assays but has a significantly lower specificity (81.6% vs. 93.8%) and PPV (55.8% vs. 75.9%) [92].

## 10. Causes of False Positive and False Negative Results

All hs-Tn assays are subject to false positive and false negative errors at the analytic level, which is a distinct process from the false positive and false negative interpretations that can arise at the clinical level. At the assay level, sources of falsely abnormal results include macrotroponin, heterophilic antibodies, autoantibodies, rheumatoid factors, fibrin interference, hemolysis, and alkaline phosphatase. Macrotroponin is a high molecular weight complex of immunoglobulin and Tn produced as a result of binding of circulating immunoglobulin with protein fragments or enzymes regardless of cardiac muscle injury. The complex can persist in circulation leading to an elevated level of Tn [93]. A study showed that macrocomplexes resulted in Tn concentration exceeding the 99th percentile, creating false positive results [94]. Interfering antibodies in patient samples, including human antianimal antibodies, heterophilic antibodies, and rheumatoid factors, can bind the assay antibodies and also result in false results [95]. Human antianimal antibodies are produced as a response to animal antibody injection for diagnostic or therapeutic purposes. Heterophilic antibodies have an affinity to animal antibodies but do not have a known exposure to antigens. While a full 40% of the general population have detectable levels of heterophilic antibodies, these do not cause falsely positive immunoassays in most patients. Rheumatoid factors, a group of autoantibodies often found in autoimmune disorders, also have cross-reactivity with animal antibodies and may interfere with immunoassays. These endogenous antibodies can either increase or decrease measured Tn levels due to reduced assay reactivity to the macro-Tn complex. In addition, fibrin clots, hemolysis, and alkaline phosphatase can elevate measured Tn levels [96,97].

**Figure 7:**
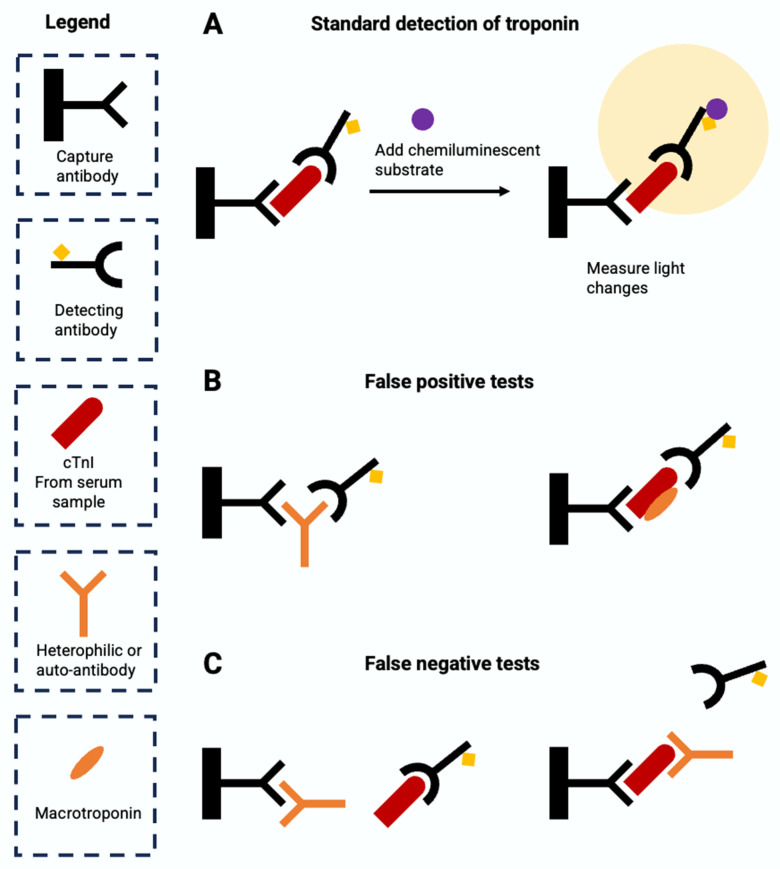
Depiction of molecular mechanisms of common sources of errors in troponin assays. (**A**). representation of chemiluminescence technology used for troponin detection. (**B**). Demonstration of false-positive result in the presence of a heterophilic antibody or autoantibody. (**C**). Demonstration of a false-negative result in the presence of macrotroponin.

At the assay level, sources of falsely normal Tn results include heterophilic or autoantibodies and analytical interference such as hyperbilirubinemia, hyperlipidemia, biotin and hemolysis [98]. Heterophilic or autoantibodies can either bind to the capture antibody, which blocks the binding of the Tn antigen to the capture antibody, or bind to the antigen, which blocks the binding of the detection antibody to the antigen. Excessive biotin also interferes with the assay by saturating the assay binding site and blocking the link between antigen and antibody [99]. In addition, pronounced hemolysis (>1 g/L), hyperbilirubinemia, and hyperlipidemia also interfere with Tn measurement [96]. The molecular mechanisms of common sources of errors in Tn assays are summarized in Figure 7.

The interference of macrotroponin, antibodies, or analytical interference often varies by assay [94]. One study measured Tn across six assays and showed Siemens hs-TnI assays had a higher percentage of patients with increased Tn measurement compared to other assays, although all assays were impacted by the existence of macrotroponin [100]. Therefore, it is important to interpret the hs-Tn test results with caution and to take into account the clinical contexts and the factors that increase the risk of error. Some common approaches to reduce the error of immunoassays include repeating the analysis using the same assay or a different assay, dilution of the sample to look for nonlinearity in the Tn concentration, blocking by neutralizing the interfering antibodies, depleting antibodies in the sample through precipitation, affinity extraction or size exclusion, and using interference assays to measure heterophilic antibodies [95]. Among those, blocking and PEG precipitation tests have been used to confirm the interference of heterophilic antibodies and macrotroponin with hs-Tn assays, respectively [93,100,101].

## 11. Conclusions

In conclusion, major advances have been made in assays for quantifying serum Tn over the last several decades. Successive generations of Tn assays demonstrate incrementally superior analytical sensitivity (e.g., LoD, LoQ) and clinical sensitivity. Because the increased clinical sensitivity comes at the cost of poorer clinical specificity, interpreting test results should involve the appropriate application of conditional probability, based on differing pre-test and post-test probability in different settings of care [74], use of contextual clinical information such as echocardiographic, electrocardiographic, and physical exam data, and use of more population-specific cutoffs to define elevated Tn such as those that take age or race into consideration [102,103]. Emerging technologies for Tn measurement include those that incorporate new nanomaterials into the biosensor, make use of in vivo measurement of biomarkers, and those that are deployed in the form of Point-of-Care Testing devices [104] and wearable Tn sensors [87]. Understanding the molecular nature of Tn and its assays is essential for the evaluation and management of patients with and without chest pain, especially as the development of advanced Tn assays promises to herald increasingly sensitive and rapid detection.

## Figures and Tables

**Table 1:** Characteristics of a test in two populations with different prevalence of disease.

In a Population with a Larger Proportion of Disease	In a Population with a Smaller Proportion of Disease
Sensitivity = static!	Sensitivity = static!
Specificity = static!	Specificity = static!
PPV = increased	PPV = decreased
NPV = decreased	NPV = increased
LR+ = static!	LR+ = static!
LR− = static!	LR− = static!
